# Comprehensive Genomic Analysis of the *CDPK* Gene Family in Pecan (*Carya illinoinensis*) and Their Potential Roles in Salt Stress Response

**DOI:** 10.3390/plants14040540

**Published:** 2025-02-10

**Authors:** Guoming Wang, Longjiao Hu, Jiyu Zhang, Min Zhai, Zhanhui Jia, Zhenghai Mo, Jiping Xuan

**Affiliations:** Jiangsu Engineering Research Center for Germplasm Innovation and Utilization of Pecan, Jiangsu Key Laboratory for the Research and Utilization of Plant Resources, Institute of Botany, Jiangsu Province and Chinese Academy of Sciences, Nanjing 210014, China; wangguoming@jib.ac.cn (G.W.); longjiaohu812@163.com (L.H.); maxzhangjy@163.com (J.Z.); zhaimin@jib.ac.cn (M.Z.);

**Keywords:** pecan (*Carya illinoinensis*), CDPK, gene expression, abiotic stress, salt stress

## Abstract

Calcium-dependent protein kinases (CDPKs) are crucial for plant development and stress responses. In this study, we performed a comprehensive genomic analysis of the *CDPK* gene family in pecan (*Carya illinoinensis*) and evaluated their potential roles in salt stress responses. A total of 31 *CiCDPK* genes were identified and classified into four subgroups through phylogenetic analysis. Structural and promoter analyses revealed conserved motifs and regulatory elements linked to stress responses. Gene duplication analysis showed that WGD and DSD events were primary drivers of *CiCDPK* expansion, shaped by purifying selection. GO and KEGG annotations highlighted roles in kinase activity, calcium binding, and signal transduction, while interaction networks suggested involvement in ROS regulation and ATP-dependent phosphorylation. Tissue-specific expression patterns indicated distinct roles of *CiCDPKs*, with *CiCDPK20* and *CiCDPK31* predominantly expressed in male flowers and seeds, respectively. Transcriptome data showed that *CiCDPKs* exhibited distinct responses to abiotic and biotic stress, highlighting their functional specialization under various conditions. qRT-PCR analysis further confirmed the involvement of 16 *CiCDPKs* in salt stress adaptation, supporting their critical roles in signal transduction pathways during salinity stress. This study provides insights into *CiCDPK* functions, offering potential applications in breeding pecan varieties with enhanced salt tolerance.

## 1. Introduction

Plants have evolved sophisticated defense strategies to adapt to both biotic and abiotic stresses, including salinity, cold, drought, and pathogen attacks [1]. These defenses are largely dependent on signaling pathways, gene regulation, and protein modifications such as phosphorylation and dephosphorylation. Protein kinases are key players in these processes, converting environmental signals into molecular and physiological responses [1]. Among them, calcium-dependent protein kinases (CDPKs) are crucial for stress signal transduction [2,3], alongside other important kinase families like mitogen-activated protein kinases (MAPKs) [4], receptor-like kinases (RLKs) [5], and SNF1-related protein kinases (SnRKs) [6,7]. Calcium ions (Ca^2^⁺) act as key cellular messengers in plants, mediating growth, development, and responses to environmental stress [8]. Fluctuations in Ca^2^⁺ levels initiate a series of physiological and biochemical responses, bolstering plants’ ability to withstand challenging environmental conditions [9]. Plants have developed a range of calcium-sensing proteins to detect and interpret Ca^2^⁺ signals, such as calmodulins (CaMs), calmodulin-like proteins (CMLs), calcineurin B-like proteins (CBLs), and CDPKs [9,10]. Among these, CDPKs function by directly translating upstream Ca^2^⁺ signals into downstream phosphorylation activities [2,3]. This dual role enables CDPKs to act as both calcium sensors and signal transducers, making them vital components of stress adaptation mechanisms.

CDPKs represent a unique family of serine/threonine kinases that integrate calcium sensing and signal transduction, enabling plants to respond to a variety of environmental and developmental signals [2,3]. CDPKs consist of four key domains: a variable N-terminal domain (VNTD) that determines subcellular localization, a serine/threonine kinase domain (PKD) responsible for phosphorylation, an autoinhibitory junction region (JD) that regulates activity, and the C-terminal calmodulin-like domain (CaMLD) contains one to four EF-hand motifs for binding Ca^2^⁺ [2,11]. This unique architecture allows CDPKs to directly integrate calcium sensing and phosphorylation, enabling them to translate calcium signals into functional responses without requiring additional interaction partners [11].

CDPKs are essential protein kinases in plant signaling, acting as mediators of Ca^2^⁺ signals critical for various physiological processes. By phosphorylating substrates such as ion channels, transcription factors, and metabolic enzymes, CDPKs regulate diverse cellular activities [2,3,12]. This enables them to play key roles in a wide range of plant functions. In plant growth and development, *CDPKs* play crucial regulatory roles. For example, several *Arabidopsis CDPKs* have been shown to regulate pollen tube growth [13]. *VvCPK4*, *5*, *6*, and *8* are strongly expressed in pollen in *Vitis vinifera* [14]. Maize Group B *CDPK* genes negatively affect seed development [15]. Banana *MaCDPK7* is involved in fruit ripening [16]. *PnCDPK1* accumulates primarily in petals and sepals of flower organs in *Pharbitis nil* [17]. *TaCPK40* in wheat negatively regulates seed dormancy but promotes seed germination [18]. In the response to abiotic and biotic stresses, *CDPKs* also play critical roles. For instance, overexpression of *AtCPK4* and *AtCPK11* in *Arabidopsis* enhances drought tolerance through improved signal transduction [19]. Similarly, overexpression of *OsCDPK7* in rice induces stress-responsive genes, improving salinity and drought tolerance [20], while *OsCPK9* positively regulates drought stress tolerance through silencing and overexpression studies [21]. In tobacco, overexpression of *ZoCDPK1* improves resistance to salt and drought stress [22]. In tomato, *SlCPK27* acts as a positive regulator of cold adaptation by modulating ABA, ROS, NO, and MPK signaling pathways [23]. Additionally, transgenic *Arabidopsis* overexpressing *ZmCPK4* exhibits enhanced drought tolerance [24]. *CDPKs* also function in biotic stress responses. In maize, *CDPK10* is implicated in defense signaling pathways [25]. In tomato, *SlCRK6* enhances resistance to *Pseudomonas syringae* and *Sclerotinia sclerotiorum* [26]. These findings underscore the versatile roles of *CDPKs* in regulating stress responses across diverse plant species.

Pecan (*Carya illinoinensis*) is an economically and nutritionally important nut crop, valued for its high-quality kernels and oil-rich seeds [27]. As a perennial tree species, pecan faces long-term exposure to abiotic and biotic stresses, significantly affecting yield and quality. Among these, salinity is one of the most detrimental, particularly in regions with high soil salinity or saline irrigation [7,28]. Understanding the molecular mechanisms of salinity tolerance in pecan is essential for improving stress resilience and sustainable production. *CDPKs*, as key regulators of calcium-mediated signaling, are promising candidates for enhancing salinity tolerance. While *CDPK* gene families have been studied in various model and crop species, no such analysis exists for pecans. This study addresses this gap by identifying and characterizing 31 *CiCDPK* genes in pecan, analyzing their phylogenetic relationships, conserved motifs, gene structures, chromosomal localization, and protein tertiary structures. Functional annotation, protein interaction predictions, and expression profiling across different tissues and stress conditions (drought, cold, salt, and Colletotrichum fioriniae infection) were conducted. Quantitative real-time PCR (qRT-PCR) further validated the involvement of *CiCDPKs* in salt stress responses. This research highlights *CiCDPK* genes as potential targets for improving stress tolerance and provides a foundation for future studies on abiotic stress adaptation and breeding stress-resilient pecan varieties.

## 2. Results

### 2.1. Identification and Phylogenetic Relationship of CiCDPK Genes

**Figure 1:**
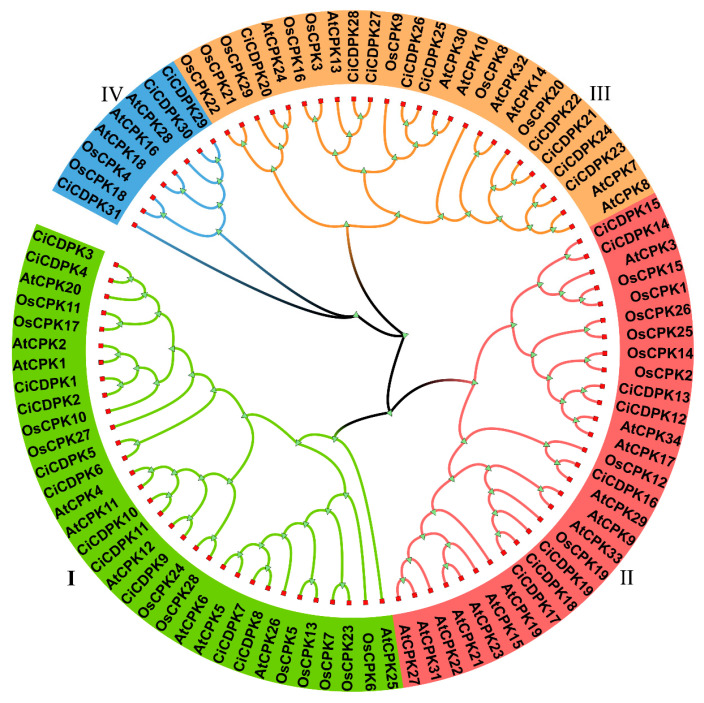
Phylogenetic analysis of *CDPKs* from *Arabidopsis*, rice, and pecan. The phylogenetic tree was constructed by MEGA 7.0 with the maximum likelihood method.

To investigate the *CiCDPK* gene family in pecan, BLASTp and HMM methods were utilized for candidate gene identification. The initial CDPK protein sequences were validated using the Pfam and SMART databases, with duplicates and incomplete sequences removed. As a result, 31 *CiCDPK* genes were identified (Supplementary Table S1). A phylogenetic tree was constructed using MEGA 7.0 to analyze evolutionary relationships. The tree was generated through the maximum likelihood (ML) method based on multiple sequence alignments of 34 *Arabidopsis thaliana* and 29 *Oryza sativa* *CDPK* genes from a previous study [2], along with the 31 pecan *CiCDPK* genes (Figure 1 and Supplementary Table S1). The results revealed that the *CiCDPK* genes clustered into four subgroups: I, II, III, and IV. Based on these clusters, the 31 *CiCDPK* genes were named *CiCDPK1-31*. Specifically, Subgroup I included 11 genes (*CiCDPK1-11*), Subgroup II contained 8 genes (*CiCDPK12-20*), Subgroup III comprised 9 genes (*CiCDPK21-28*), and Subgroup IV included 3 genes (*CiCDPK29-31*). Genes within the same subgroup are likely to have similar functions.

### 2.2. Analysis of Conserved Motifs and Gene Features

**Figure 2:**
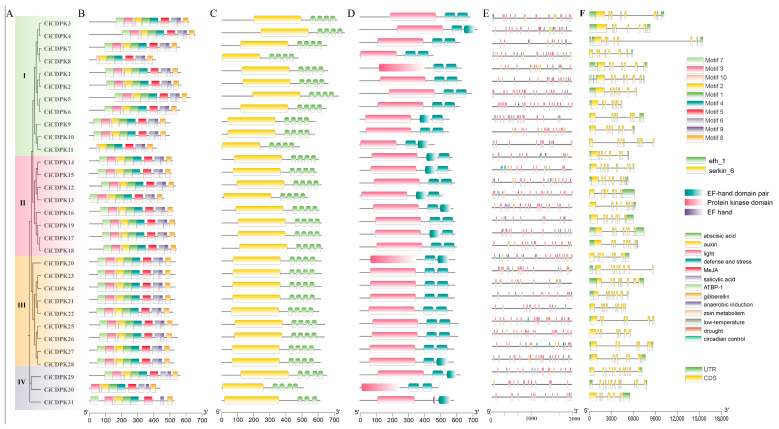
Phylogenetic and structural characteristics of CiCDPK family members in pecan. (**A**) Phylogenetic tree. (**B**) Motif composition. (**C**) Conserved domain by SMART. (**D**) Conserved domain by Pfam. (**E**) Cis-regulatory elements identified in the promoter 2000 bp regions of the *CiCDPKs*. (**F**) Exon, intron, and UTR structures. The lengths of genes and proteins are represented on the scale provided at the bottom.

To analyze the structural features of the 31 *CiCDPKs* in pecan, motif and domain composition were assessed using the MEME program and the Pfam and SMART databases. Additionally, promoter regions, exon-intron structures, and UTRs were analyzed using PlantCARE and GSDS 2.0 (Figure 2). MEME analysis identified 10 conserved motifs across most CiCDPKs, except for CiCDPK30 and CiCDPK31 in Subgroup IV (Figure 2B). All CiCDPKs were found to contain the conserved domains Ser_kin_6 and four EF-hand domains (EFh_1), except CiCDPK31, which has three EF-hand domains, based on SMART analysis (Figure 2C). Similarly, all CiCDPKs were confirmed to have a protein kinase domain and two EF-hand domain pairs, with the exception of CiCDPK31, which has one EF-hand domain pair and one additional EF-hand (Figure 2D). This is consistent with observations in *Arabidopsis*, where all CDPKs except CPK25 possess four EF hands, while CPK25 has a truncated C-terminal region containing only one EF hand [2]. Promoter analysis using PlantCARE revealed that *CiCDPKs* are enriched with cis-regulatory elements related to transcription factors, light responsiveness, plant hormones, circadian rhythms, anaerobic induction, salicylic acid, and stress responses (Figure 2E). Most *CiCDPKs* contain 7–8 exons, while *CiCDPK29* and *CiCDPK30* from Subgroup IV have the highest number of introns, with 11–12, showing highly similar gene structures (Figure 2F). The isoelectric points (pI) of *CiCDPKs* range from 4.66 to 9.28, and their molecular weights (MW) range from 46.05 to 72.85 kDa (Supplementary Table S1). Subcellular localization predictions using the Wolf PSORT tool suggest that CiCDPKs are distributed across various organelles, including the vacuole, chloroplast, nucleus, cytoplasm, peroxisome, and mitochondrion (Supplementary Table S1).

### 2.3. Analysis of Protein Tertiary Structures

**Figure 3:**
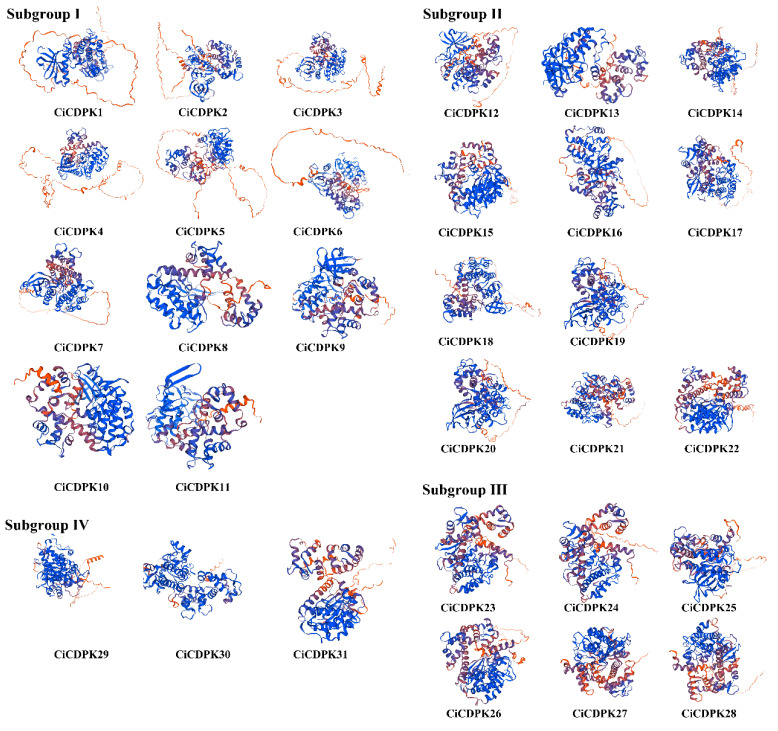
Predicted tertiary structures of CiCDPK proteins generated using the AlphaFold2 method. The models are displayed with a rainbow color scheme, representing the N-terminus to the C-terminus.

Protein tertiary structures provide essential insights into their functional and structural properties. To better understand the structural characteristics of CiCDPK proteins, their tertiary structures were predicted using AlphaFold2, an advanced machine learning-based tool capable of achieving near-experimental accuracy solely from primary amino acid sequences. For comparative purposes, template proteins identified through SWISS-MODEL were used to model the tertiary structures of the CiCDPKs (Figure 3). The predicted structures of the 31 CiCDPK proteins displayed a conserved architecture consisting of α-helices and β-pleated sheets, forming coiled and stranded regions. Notably, subtle structural variations were observed between different subgroups of CiCDPKs, which may correlate with their functional diversity. These features highlight key hydrophobic interactions critical for protein-protein complex formation and the stabilization of protein structure. The rainbow-colored models further illustrated structural patterns across the N-terminus to the C-terminus, emphasizing conserved regions and subgroup-specific differences.

### 2.4. Gene Localization, Duplication Patterns, and Ka/Ks Analysis of CiCDPKs

**Figure 4:**
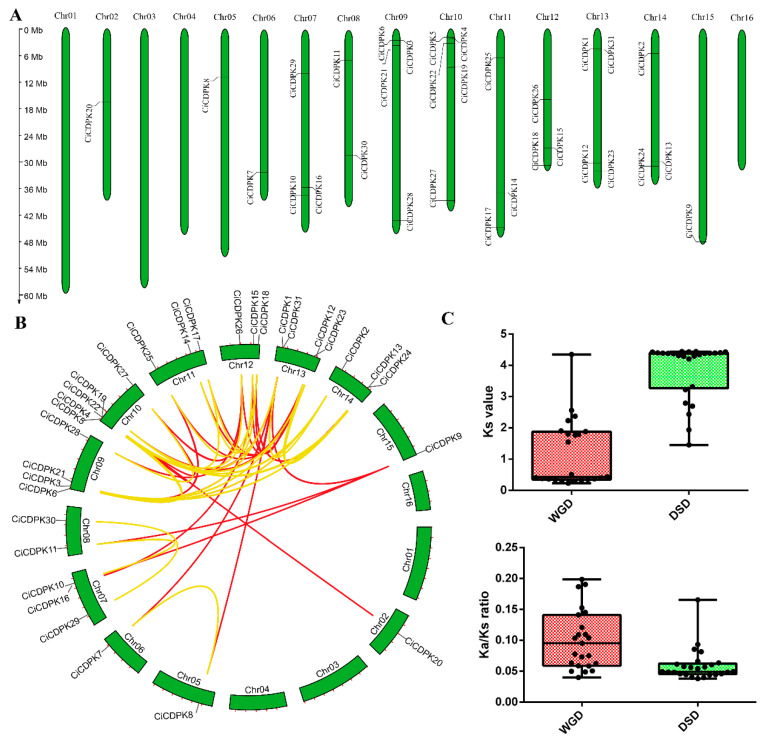
Distribution, duplication events, and *Ka/Ks* of the *CiCDPK* family genes. (**A**) Chromosome locations. (**B**). Gene duplication modes, with yellow and red lines representing WGD (whole-genome duplication) and DSD (dispersed duplication), respectively. (**C**) Distribution of *Ks* and *Ka/Ks* values for WGD and DSD duplication events.

Chromosomal localization analysis revealed that the 31 *CiCDPK* genes are unevenly distributed across 12 chromosomes, with no genes mapped to chromosomes 01, 03, 04, and 16 (Figure 4A). To explore the impact of different gene duplication mechanisms on the expansion of the *CiCDPK* gene family in pecan, five duplication types, including whole-genome duplication (WGD), tandem duplication (TD), proximal duplication (PD), transposed duplication (TRD), and dispersed duplication (DSD), were analyzed. Among these, only WGD and DSD events were identified (Figure 4B and Supplementary Table S2). These duplication modes highlight the key processes contributing to the diversification and expansion of the *CiCDPK* gene family, with WGD and DSD being the primary drivers.

To assess evolutionary divergence and selection pressures on the *CiCDPK* genes, the ratios of nonsynonymous (*Ka*) to synonymous (*Ks*) substitutions (*Ka/Ks*) were calculated. A *Ka/Ks* ratio greater than 1 indicates positive selection, a ratio of 1 suggests neutral evolution, and a ratio below 1 reflects negative (purifying) selection [29]. Remarkably, all *Ka/Ks* ratios for WGD and DSD paralogous gene pairs were found to be less than 1 (Figure 4C and Supplementary Table S2), suggesting that purifying selection has predominantly shaped the evolution of the *CiCDPK* gene family in pecan.

### 2.5. Prediction of miRNAs Targeting CiCDPK Genes

**Figure 5:**
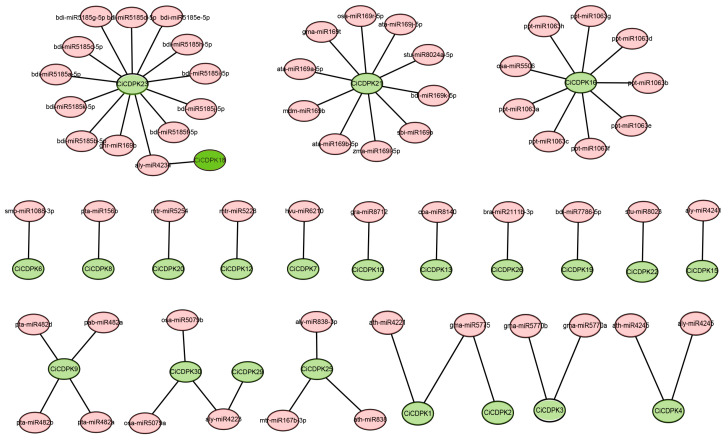
Network map of projected miRNA-target interactions with *CiCDPK* genes. The green ellipses represent *CiCDPK* genes, while the pink ellipses indicate the predicted miRNAs.

MicroRNAs (miRNAs) are key regulators of gene expression, often influencing post-transcriptional processes, including stress adaptation and plant development. To explore the regulatory roles of miRNAs on *CiCDPK* genes, target sites were predicted using the psRNATarget database. The analysis identified several miRNAs interacting with specific *CiCDPK* family members, with varying degrees of target site distribution across the gene family (Figure 5). A total of 27 miRNAs were identified to target 23 *CiCDPK* genes. Notably, *CiCDPK16*, *CiCDPK21*, and *CiCDPK23* were targeted by more than nine miRNAs originating from different species, highlighting their potential as key regulatory hubs. Conversely, certain miRNAs were found to target multiple *CiCDPK* genes, such as alt-miR4234, alt-miR4223, and gma-miR5775b, suggesting their broad influence on the regulation of the *CiCDPK* gene family. The findings suggest that certain miRNAs target multiple *CiCDPK* genes, while others exhibit high specificity. Many of the identified miRNAs are associated with stress-related pathways, indicating their potential role in modulating *CiCDPK* functions during abiotic stress responses such as salinity, drought, and cold.

### 2.6. Functional Annotation and Prediction of Protein-Protein/Chemical Interactions

Gene Ontology (GO) enrichment analysis was performed for the 31 *CiCDPK* genes, and all were enriched in terms related to molecular functions (MF) (Supplementary Table S3). The key GO terms included GO:0004672 (protein kinase activity), GO:0004713 (protein tyrosine kinase activity), GO:0005509 (calcium ion binding), GO:0005524 (ATP binding), and GO:0006468 (protein phosphorylation). These findings suggest that the *CiCDPK* genes primarily function as kinases involved in phosphorylation processes and calcium signaling, which are critical for cellular signal transduction and regulatory mechanisms. For the KEGG pathway analysis, most *CiCDPK* genes were annotated as K12132 (eukaryotic-like serine/threonine-protein kinase [EC:2.7.11.1]), indicating their roles as serine/threonine-protein kinases involved in phosphorylation and signal transduction pathways. Notably, *CiCDPK14* and *CiCDPK15* were annotated as K08884 (bacterial serine/threonine-protein kinase [EC:2.7.11.1]), which is typically associated with prokaryotic-like kinases (Supplementary Table S3). This unique annotation suggests potential functional divergence for these two genes, possibly reflecting their adaptation to specific regulatory or stress-response pathways. These results provide insights into the functional diversity and evolutionary specialization of the *CiCDPK* gene family in pecan.

**Figure 6:**
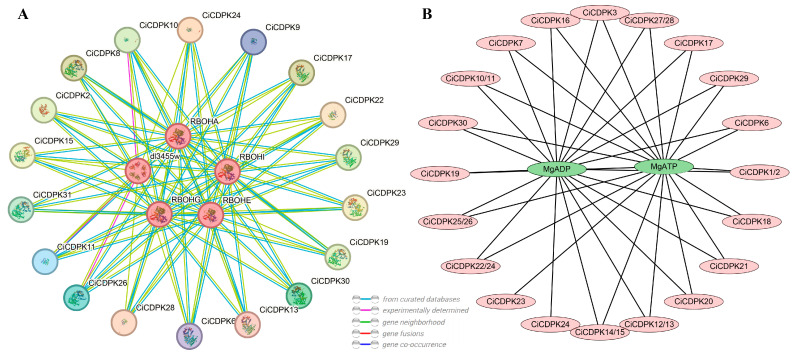
Interaction analysis of CiCDPK proteins using predicted AtCDPK orthologs by STRING and STITCH. (**A**) Predicted protein–protein interaction network, with interacting proteins highlighted in red circles. (**B**) Protein–chemical interaction network, where CiCDPK proteins are represented by pink ellipses, and interacting chemicals are shown as green ellipses. The interaction networks were visualized using Cytoscape.

Protein–protein and protein–chemical interaction networks of CiCDPK proteins were constructed using predicted orthologs from Arabidopsis thaliana through STRING and STITCH analysis (Figure 6). The protein–protein interaction network identified five potential interacting proteins: dl3455w, RBOHI, RBOHE, RBOHA, and RBOHG (Figure 6A). Notably, these proteins are associated with reactive oxygen species (ROS) production and stress response, suggesting that CiCDPKs may play a key role in regulating ROS-mediated signaling pathways in pecan. In the protein–chemical interaction network, CiCDPK proteins were predicted to interact with MgADP and MgATP, which are essential cofactors for kinase activity (Figure 6B). These interactions highlight the involvement of CiCDPKs in ATP-dependent phosphorylation processes, further supporting their role in signal transduction and energy regulation. Together, these findings provide insights into the functional roles of CiCDPKs, particularly in stress signaling and energy metabolism in pecan.

### 2.7. Expression Pattern of CiCDPK Family Genes in Different Pecan Tissues

**Figure 7:**
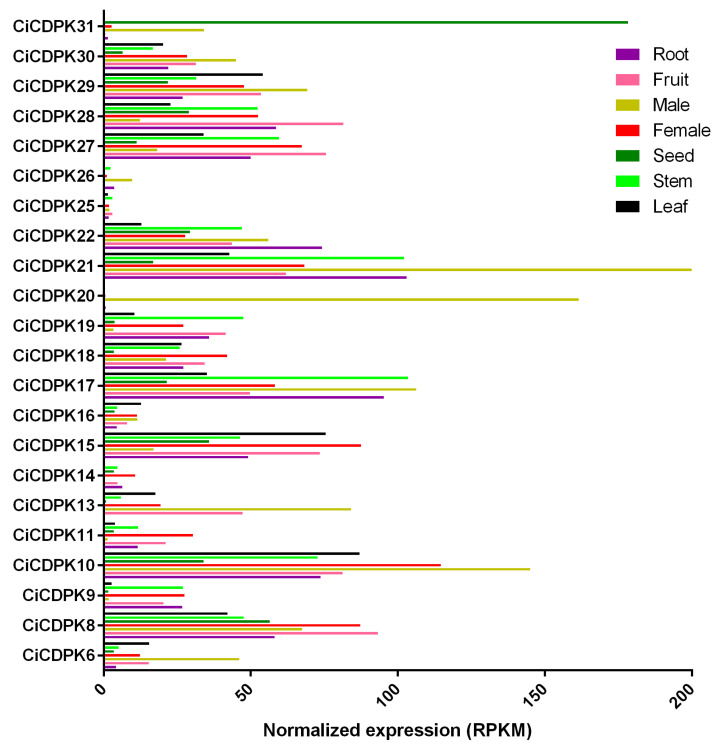
Expression patterns of *CiCDPK* genes in different pecan tissues based on RNA-seq. Expression levels are presented as average RPKM (reads per kilobase of exon model per million mapped reads) across root, leaf, seed, male and female flowers, and fruit.

The expression patterns of *CiCDPK* genes were analyzed across various pecan tissues, including root, leaf, seed, male and female flowers, and fruit (Figure 7 and Supplementary Table S4). Notably, nine genes (*CiCDPK1*, *2*, *3*, *5*, *6*, *7*, *12*, *23*, *24*) showed no detectable expression in any of the tissues examined, indicating their potential roles may be restricted to specific conditions or developmental stages not represented in the dataset. *CiCDPK25* and *CiCDPK26* exhibited extremely low expression levels across all tissues, suggesting limited functional activity or regulation at the post-transcriptional level. Interestingly, *CiCDPK20* showed tissue-specific expression, with a high level of expression exclusively in male flowers, indicating its potential involvement in male-specific developmental processes or signaling pathways. Similarly, *CiCDPK31* demonstrated a high expression level specifically in seeds, highlighting its possible role in seed development or metabolic regulation. These distinct expression patterns suggest functional specialization of *CiCDPK* family members in pecan and provide insights into their potential roles in tissue-specific biological processes.

### 2.8. Expression Patterns of CiCDPK Genes in Response to Abiotic and Biotic Stresses

**Figure 8:**
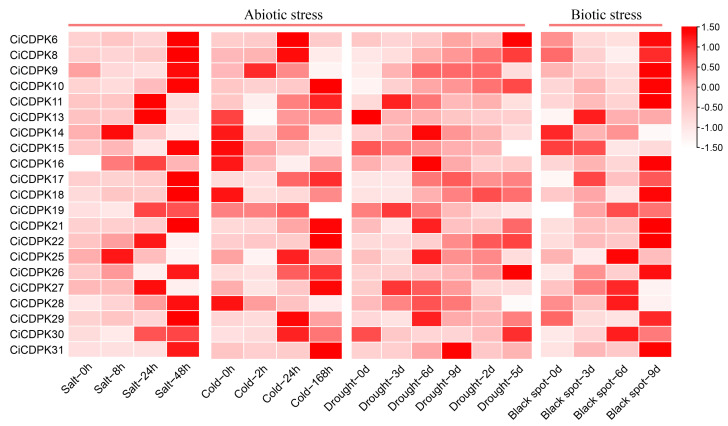
Expression patterns of *CiCDPK* genes under abiotic and biotic stresses. Expression levels of *CiCDPK* genes were analyzed under salt, drought, cold, and *Colletotrichum fioriniae* infection, shown as average RPKM values.

To further validate the involvement of *CiCDPK* genes in responses to abiotic and biotic stresses, transcriptome analysis was performed using publicly available data [28,30,31]. After excluding 10 genes with no detectable expression, heatmap analysis showed that 21 *CiCDPK* genes responded to drought, cold, salt, and *Colletotrichum fioriniae* infection (Figure 8 and Supplementary Table S5). Under salt stress, *CiCDPK4* showed a sharp increase at 48 h, reaching the highest expression level, while *CiCDPK14* displayed a moderate upregulation at 8 h but decreased significantly afterward. During cold stress, *CiCDPK10* and *CiCDPK21* demonstrated consistent upregulation, particularly at 24 h and 168 h, indicating their potential roles in prolonged cold stress adaptation. In response to drought stress, *CiCDPK10*, *CiCDPK22*, and *CiCDPK29* showed significantly elevated expression levels, with *CiCDPK22* peaking at 5 d. This suggests their involvement in sustained drought response mechanisms. Similarly, *CiCDPK8* and *CiCDPK29* exhibited strong induction under *Colletotrichum fioriniae* infection, peaking at 9 d, implying their roles in biotic stress resistance. These results indicate that *CiCDPK* genes exhibit distinct expression patterns under various stresses, reflecting their functional specialization in pecan’s abiotic and biotic stress responses.

### 2.9. qRT-PCR Analysis of Genes in Response to Salt Stress

**Figure 9:**
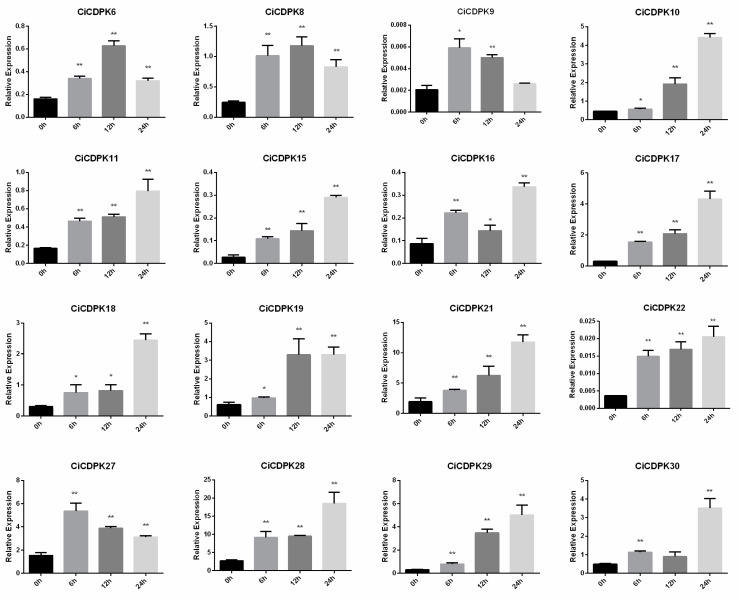
qRT-PCR analysis of *CiCDPK* genes under NaCl treatment in pecan seedlings. Standard errors were calculated, and significance levels were determined using Student’s *t*-test. Single and double asterisks indicate significant differences at * *p* < 0.05 and ** *p* < 0.01, respectively.

To further examine the roles of *CiCDPK* genes in response to salt stress, qRT-PCR analysis was conducted. A total of 16 *CiCDPK* genes were selected based on transcriptome data, excluding non-expressed and low-abundance genes. These genes were analyzed at four time points (0, 6, 12, and 24 h) following NaCl treatment, revealing distinct expression patterns (Figure 9). Most genes showed consistent upregulation under salt stress. Specifically, *CiCDPK10*, *11*, *15*, *17*, *21*, *22*, and *29* exhibited sustained upregulation throughout the treatment period. *CiCDPK4* and *CiCDPK8* displayed continuous upregulation up to 12 h, followed by a decline in expression. *CiCDPK9* and *CiCDPK27* showed peak expression at 6 h, with subsequent downregulation. Interestingly, *CiCDPK16* and *CiCDPK30* demonstrated a dynamic expression trend, with initial upregulation, followed by downregulation, and then a subsequent increase. These findings indicate that *CiCDPK* genes respond to salt stress through diverse and time-specific expression patterns, suggesting their involvement in salinity adaptation in pecan.

## 3. Discussion

*CDPK* genes, widely present in photosynthetic organisms, serve as critical calcium signal decoders, regulating plant growth, development, hormone signaling, abiotic stress responses, and innate immunity [1,2,3]. *CDPK* genes have been extensively studied in various plants, including *Arabidopsis*, rice, maize, wheat, soybean, cotton, cucumber, banana, tomato, grape, and poplar [2,32,33,34]. However, limited information is available regarding *CDPKs* in pecan. In this study, 31 *CiCDPK* genes were identified using BLASTp and HMM methods. Phylogenetic analysis grouped these genes into four subgroups (I, II, III, and IV), consistent with *CDPK* classifications in *Arabidopsis* and rice (Figure 1) [2]. The number of *CDPKs* in each subgroup was comparable to those in *Arabidopsis* and rice, indicating no significant evolutionary expansion of the *CDPK* family in pecan. *CDPKs* were found to contain complete sequences, including a variable N-terminal domain, a C-terminal domain, a kinase domain for target phosphorylation, and EF-hand motifs for calcium binding [2,11]. A total of 30 *CiCDPKs* exhibited a conserved structure with a protein kinase domain and four EF-hand motifs (arranged as two pairs) (Figure 2), which enhance calcium-binding capacity and structural stability. However, *CiCDPK31* was identified with only three EF-hand motifs, a feature also observed in *AtCPK25*, which has a truncated C-terminal region containing a single EF-hand motif [2]. Similarly, reduced EF-hand motifs, as reported in *FaCDPK3* and *FaCDPK8*, were associated with significantly lower calcium-binding sensitivity and diminished calcium-induced conformational changes, potentially affecting kinase activity [35]. The conserved clustering across subgroups indicates an evolutionary preservation of functional roles, with genes within the same cluster likely performing similar biological functions.

Gene duplication events are vital for genome evolution, allowing organisms to acquire new functions and adapt to changing environments [36]. These duplications result from the replication and retention of specific genetic sequences, leading to multiple copies of a gene or gene region [37]. Various mechanisms, such as WGD, DSD, transposed duplication (TRD), proximal duplication (PD), and tandem duplication (TD), contribute to this process, driving functional innovation and diversification [37]. This study showed that WGD and DSD events were the main drivers of *CiCDPK* family expansion in pecan (Figure 3). WGD and DSD events are key drivers of eukaryotic genome evolution, fostering the emergence of novel gene functions and advancing genetic innovation [37,38]. For instance, many gene families, such as Hsfs (heat shock transcription factors) [39], F-box families [40], PYL (pyrabactin resistance 1-like) [41], MAPKKK (mitogen-activated protein kinase kinase kinase) [4], and PP2C (Type 2C protein phosphatase) [42], have primarily expanded through WGD and DSD events. The Ka/Ks analysis revealed that all duplicated gene pairs underwent purifying selection (Figure 3), indicating functional conservation during evolution. These findings suggest that the *CiCDPK* gene family has evolved through duplication events while maintaining its core functions, enabling pecan to adapt to various environmental challenges.

The functional diversity of *CDPKs* has been extensively studied, highlighting their pivotal roles in plant growth, development, and responses to various environmental stresses through complex regulatory mechanisms. Tissue-specific expression patterns, such as the high expression of *CiCDPK20* in male flowers and *CiCDPK31* in seeds, highlight their specialized roles in reproductive development (Figure 7). GO and KEGG analyses confirmed that *CiCDPKs* primarily function in kinase activity, calcium signaling, and phosphorylation processes. Interaction networks suggested potential roles of *CiCDPKs* in regulating reactive oxygen species (ROS) production, supported by predicted interactions with ROS-related proteins (RBOHs) (Figure 6). However, these interactions require further experimental validation. Moreover, protein–chemical interactions with MgADP and MgATP indicate their involvement in ATP-dependent phosphorylation, critical for stress signaling and energy regulation (Figure 6). These results underline the functional versatility of *CiCDPKs* in pecan’s stress response mechanisms. *CDPKs* are known to play critical roles in calcium-mediated stress responses, particularly under salt stress. For instance, *AtCPK11* and *AtCPK4* in *Arabidopsis* enhance drought tolerance [19], while *OsCDPK7* in rice promotes salinity and drought resistance [20]. In this study, transcriptome analyses demonstrated the involvement of *CiCDPKs* in salt stress, with several genes showing significant changes in expression under NaCl treatment (Figure 8). 16 *CiCDPKs* displayed significant upregulation under salt stress by qRT-PCR (Figure 9). Moreover, integrating *CiCDPK* candidate genes into pecan breeding programs through marker-assisted selection or gene editing technologies could provide new strategies for developing salt-tolerant cultivars. These findings suggest that *CiCDPKs* may function as key regulators in the adaptation of pecan to salinity, similar to their roles in other species. Further functional studies are necessary to confirm their specific contributions to salt stress tolerance.

## 4. Materials and Methods

### 4.1. Identification and Phylogenetic Analysis of CDPK Genes in Pecan

Genomic data for the ‘Pawnee’ variety of pecan were obtained from the Phytozome database (http://phytozome-next.jgi.doe.gov/, accessed on 2 December 2024) [43]. The identification of CDPK proteins was initially carried out using the BLASTP and Hidden Markov Model (HMM) programs. To refine the search, protein kinase domains (PF00069) and EF-hand domains (PF13499) were downloaded from the InterPro database (https://www.ebi.ac.uk/interpro/, accessed on 2 December 2024). The HMMER 3.0 program was then utilized to detect CDPK sequences, applying the default threshold for domain identification. A TBLASTN search (version 2.2.26, Bethesda, Rockville, MD, USA) was performed against the ‘Pawnee’ genome using the 34 known CDPK proteins of *Arabidopsis thaliana* [2] as queries, with an E-value cutoff of 1 × e^−10^. For further verification, candidate CDPK protein sequences were subsequently validated through analysis in the Pfam and SMART databases available via InterPro (https://www.ebi.ac.uk/interpro/, accessed on 4 December 2024). Phylogenetic trees were generated using MEGA 7.0 [44] based on the full-length CDPK protein sequences from pecan. The maximum likelihood method was applied, utilizing a pairwise distance matrix calculated from the amino acid sequences. Bootstrap analysis was performed with 1000 replications to assess the reliability of the tree topology. The phylogenetic tree was further enhanced and edited using the iTOL platform (https://itol.embl.de/, accessed on 4 December 2024).

### 4.2. Characterization and Structure Analysis of CiCDPKs

The isoelectric point (pI) and molecular weight (MW) of the proteins were calculated using the Sequence Manipulation Suite (SMS 2.0) (http://www.bioinformatics.org/sms2/index.html/, accessed on 4 December 2024). Subcellular localization was predicted using the Wolf PSORT tool (https://wolfpsort.hgc.jp/, accessed on 4 December 2024). The MEME tool was employed to identify conserved motifs in CiCDPK proteins through Motif Elicitation. Pfam and SMART motif analyses were performed via the InterPro database (https://www.ebi.ac.uk/interpro/, accessed on 4 December 2024). Cis-acting elements in the 2000 bp upstream promoter region of the CiCDPKs sequences were predicted using PlantCARE (http://bioinformatics.psb.ugent.be/webtools/plantcare/html/, accessed on 4 December 2024). Gene structure analysis, including intron and exon regions of CiCDPKs, was conducted using the GENE Structure Display Server (GSDS 2.0) (http://gsds.gao-lab.org/, accessed on 4 December 2024). The results were visualized with TB-tools software v2.149 [45]. The 3D structure of the CiCDPKs proteins was predicted using the SWISS-MODEL server (https://swissmodel.expasy.org, accessed on 4 December 2024).

### 4.3. Gene Location and Duplication Events Analysis of CiCDPKs

Gene chromosomal localization was performed using MG2C (http://mg2c.iask.in/mg2c_v2.1/, accessed on 8 December 2024). Synteny analysis of the pecan genome, including the identification of homologous gene pairs and their locations, was conducted using PGDD, MCScanX, and DupGen_finder, as described in [7]. To assess the evolutionary relationships of duplicate gene pairs, Ka/Ks ratios, nonsynonymous (Ka), and synonymous (Ks) substitution rates were calculated using KaKs_Calculator 2.0 [46].

### 4.4. miRNA Target Predictions

MicroRNA target sites for *CiCDPKs* were identified using the psRNATarget database (https://www.zhaolab.org/psRNATarget/, accessed on 8 December 2024). The interaction network between miRNAs and *CiCDPKs* was constructed using Cytoscape (v3.10) (https://cytoscape.org/download.html, accessed on 8 December 2024).

### 4.5. Functional Annotation Evaluation

The functional annotation of *CiCDPKs* was performed using the Gene Ontology (GO) database (http://www.geneontology.org/, accessed on 10 December 2024) and the Kyoto Encyclopedia of Genes and Genomes (KEGG) database (http://www.genome.jp/kegg/, accessed on 10 December 2024).

### 4.6. Protein–Protein/Chemical Interaction Analysis

Protein–protein interactions were predicted using STRING (https://cn.string-db.org/, accessed on 10 December 2024), while protein–chemical interactions were analyzed using STITCH (http://stitch.embl.de/, accessed on 10 December 2024). Interaction networks were visualized and constructed using Cytoscape (v3.10).

### 4.7. Transcriptome Expression Patterns Analysis of CiCDPKs

RNA-seq datasets representing various pecan tissues, such as fruits, roots, female and male flowers, leaves, and seeds, were obtained from the National Center for Biotechnology Information (NCBI) database under BioProject ID PRJNA799663 [30]. Additionally, RNA-seq reads from pecan seedlings subjected to drought, cold, salt stress, and *Colletotrichum fioriniae* infection were downloaded with accession numbers PRJNA743302, PRJNA767196, PRJNA589673, and PRJNA894234, respectively [28,30,31]. The raw sequencing reads were processed to remove poly (A/T) tails, adapter sequences, and low-quality reads before being aligned to the reference genome using HISAT2. Transcript abundance was quantified with feature counts, and gene expression levels were calculated as RPKM. Heatmaps illustrating the expression patterns of *CiCDPK* genes were generated using TBtools software v2.149 [45].

### 4.8. Plant Materials Treatment and qRT-PCR Analysis

Pecan ‘Pawnee’ seedlings were cultivated at the Institute of Botany, Jiangsu Province, and Chinese Academy of Sciences, Nanjing, under controlled growth chamber conditions with a 16/8 h light/dark cycle. Three-month-old seedlings were treated with 200 mM NaCl to induce salinity stress. Leaves from both treated and control seedlings were collected at 0, 6, 12, and 24 h, quickly frozen in liquid nitrogen, and stored at −80 °C for further use. Total RNA was extracted using a Tiangen RNA Isolation Kit (Beijing, China) and reverse-transcribed into first-strand cDNA using a TransScript One-Step gDNA Removal and cDNA Synthesis SuperMix (TransGen, Beijing, China). Specific primers for *CiCDPK* genes were designed using the Primer-BLAST tool (http://www.ncbi.nlm.nih.gov/tools/primer-blast/, accessed on 12 December 2024), with serine/threonine-protein phosphatase-1 (*PP1*) used as the reference gene [47]. A detailed list of primers is provided in Table S6. Quantitative real-time PCR (qRT-PCR) was performed using a LightCycler 480 SYBR Green I Master kit (Roche, USA) with three biological replicates. The relative expression levels were calculated using the 2^−ΔΔCt^ method.

## 5. Conclusions

Based on the comprehensive genomic analysis of the *CiCDPK* gene family in pecan, this study provides valuable insights into the molecular mechanisms underlying salt stress responses. The identification and functional characterization of 31 *CiCDPK* genes highlight their potential roles in regulating stress-related signaling pathways, particularly in salinity tolerance. Our findings offer a strong foundation for future studies aimed at improving stress resilience in pecans through targeted breeding strategies.

## Data Availability

The data presented in this study are available in the graphs and tables provided in the manuscript.

## Supplementary Materials

The following supporting information can be downloaded at: https://www.mdpi.com/article/10.3390/plants14040540/s1, Supplementary Table S1: Gene features of 31 *CiCDPK* genes in pecan; Supplementary Table S2: Gene duplication events identified and Ka, Ks and Ka/Ks analysis in *CiCDPK* gene family; Supplementary Table S3: GO and KEGG annotation of *CiCDPKs* in pecan; Supplementary Table S4: RPKM data in different tissues of pecan; Supplementary Table S5: RPKM under various biotic/abiotic stress of pecan seeding; Supplementary Table S6: Primer sequences used for qRT-PCR test.

## Abbreviations

The following abbreviations are used in this manuscript: Ca^2^⁺: Calcium Ions; CaMLD: Calmodulin-Like Domain; CaMs: Calmodulins; CBLs: Calcineurin B-Like Proteins; CDPKs: Calcium-Dependent Protein Kinases; CMLs: Calmodulin-Like Proteins; DSD: Dispersed Duplication; EF-hand: Calcium-Binding Helix-Loop-Helix Motif; GO: Gene Ontology; GSDS: Gene Structure Display Server; HMM: Hidden Markov Model; KEGG: Kyoto Encyclopedia of Genes and Genomes; MAPKs: Mitogen-Activated Protein Kinases; ML: Maximum Likelihood; MW: Molecular Weight; pI: Isoelectric Point; PD: Proximal Duplication; PKD: Protein Kinase Domain; PP2C: Type 2C Protein Phosphatase; PYL: Pyrabactin Resistance 1-Like; RLKs: Receptor-Like Kinases; RNA-seq: RNA Sequencing; RPKM: Reads Per Kilobase of Exon Model Per Million Reads Mapped; ROS: Reactive Oxygen Species; SnRKs: SNF1-Related Protein Kinases; TD: Tandem Duplication; TRD: Transposed Duplication; UTR: Untranslated Region; WGD: Whole-Genome Duplication.
